# Rapeseed Oil-Based Collagen–Agar Emulsion Gel as an Alternative Fat Raw Material in Model Liver Pâté-Type Meat Systems

**DOI:** 10.3390/foods15152609

**Published:** 2026-07-25

**Authors:** Iwona Szymańska, Edyta Kowalczyk, Aneta Cegiełka, Marta Chmiel, Lech Adamczak, Tomasz Florowski

**Affiliations:** Department of Food Technology and Assessment, Institute of Food Sciences, Warsaw University of Life Sciences–SGGW, 159C Nowoursynowska Street, 02-776 Warsaw, Poland; edyta.kowalczyk12@gmail.com (E.K.); marta_chmiel@sggw.edu.pl (M.C.); lech_adamczak@sggw.edu.pl (L.A.); tomasz_florowski@sggw.edu.pl (T.F.)

**Keywords:** emulsion gel, animal fat replacement, collagen, agar, rapeseed oil, meat model system, liver pâté

## Abstract

This study assessed the effect of replacing animal fat (scalded pork jowl) with a rapeseed oil-based collagen–agar emulsion gel (CAEG) on the quality parameters of model liver pâté-type meat systems. Model pâté batters and baked pâtés were prepared with fat replacement levels of 0, 25, 50, 75, and 100%. The samples were analyzed for pH, color parameters, apparent viscosity, cooking yield, water activity, chemical composition, energy value, TBARS index, shear force, and structure. The use of CAEG as a partial replacer for pork jowl had no significant impact on the pH or color parameters (L*, a*, b*) of the pâté batters. It also did not affect the pH, water activity, cooking yield, or color parameters (L*, a*) of the baked pâtés. Increasing pork jowl replacement reduced pâté batter viscosity and baked product shear force, accompanied by a less fibrous protein network structure, as observed by microscopic analysis. At ≥50% replacement, significant reductions in fat, saturated fatty acids (SFA), and protein content, energy value, and TBARS levels were observed. However, excessive replacement of pork jowl with CAEG (≥75–100%) negatively affected texture, resulting in softer and less cohesive structures. Overall, the partial replacement of pork jowl with a rapeseed oil-based CAEG can improve nutritional and oxidative properties of liver pâté-type systems, while maintaining acceptable technological quality up to 50% replacement.

## 1. Introduction

The quality of processed meat products is influenced by multiple factors, among which recipe composition and processing technology are particularly important, and vary depending on the product type [1,2,3]. Edible offal-processed meat products, such as pâtés, are widely consumed in Europe and other regions worldwide. They are appreciated for their characteristic flavor and texture [4,5,6]. Animal fats play a key role in determining the sensory and structural properties of emulsion-type meat products [7,8]. However, these products are often considered nutritionally less favorable due to their relatively high fat content (25–50%) [9,10,11], high levels of saturated fatty acids (SFA), and the presence of cholesterol [9,12,13,14]. Excessive consumption of such compounds is associated with an increased risk of obesity, metabolic disorders, and cardiovascular diseases [15,16].

In response to increasing consumer awareness and current nutritional recommendations, there is growing interest in reformulating meat products by replacing animal fat with alternative ingredients [17,18,19]. The effectiveness of such strategies depends on the type and level of fat replacers used, as well as the targeted nutritional and technological outcomes. The main objective is not only to reduce total fat content but also to improve the fatty acid profile of the final product, while minimizing undesirable effects on technological properties, microbiological safety, and shelf life [20,21,22,23,24].

One of the main approaches in meat product reformulation is the partial replacement of animal fat with lipid systems based on vegetable oils rich in unsaturated fatty acids [21,23]. Rapeseed oil is of particular interest due to its favorable fatty acid composition, high content of monounsaturated fatty acids, and the presence of α-linolenic acid from the n-3 group [25]. However, direct incorporation of liquid oils is limited by their low oxidative stability and lack of structure-forming ability typical of animal fats [26,27]. Therefore, increasing attention has been given to structured lipid systems that immobilize oil within a three-dimensional biopolymer network, forming semi-solid structures resembling conventional animal fats [28,29].

Such strategies include integrating emulsification and hydrogelation techniques. Emulsion gels (gelled emulsions) are typically based on oil-in-water systems in which the lipid phase is dispersed in a continuous aqueous phase and subsequently structured via gelation using biopolymers, mainly proteins and polysaccharides. These systems have demonstrated strong potential as animal fat mimetics, supporting the development of healthier meat products [27,30,31,32,33]. Research has focused on synergistic interactions between proteins (animal- and plant-derived) and polysaccharides (e.g., starch, cellulose derivatives, gums, dietary fibers, and carrageenan), which act as emulsifying, thickening, and/or gelling agents [34,35,36,37,38]. Their application may improve lipid profiles while maintaining technological functionality; however, challenges remain regarding structural stability and texture [23,39,40,41]. Thus, the development of systems compatible with conventional meat-processing technologies remains necessary [42].

Collagen, one of the most widely used food proteins, is typically obtained from skin, cartilage, bones, tendons, intramuscular connective tissue, and blood vessels of land animals, primarily cows and pigs [43,44]. Depending on its origin and processing conditions, collagen exhibits diverse functional properties related to gelling behavior (e.g., water-holding capacity, thickening, and gel network formation), as well as surface behavior (e.g., adhesiveness, cohesiveness, film-forming ability, and the stabilization of emulsions and foams) [45,46,47]. These properties are particularly relevant as functional ingredients in meat processing [48,49,50,51].

Among food polysaccharides, agar (a red seaweed-derived hydrocolloid composed mainly of agarose and agaropectin) has attracted considerable attention in the food industry [52]. Even at low concentrations (e.g., 0.2%), it forms firm thermoreversible gels with high structural stability due to hydrogen bonding between agarose chains, without the need for additional gelling agents [53]. Agar gels are generally considered relatively flavor-neutral; however, when incorporated into food products, they may absorb aroma and color compounds from the matrix, potentially enhancing the product characteristics [54]. Moreover, it has been applied in the structuring of vegetable oils in Pickering emulsions [55], hydrogelled oleogel systems [56], and composite emulsion gels [39,57,58].

The combination of collagen and agar in co-gel systems may offer synergistic effects due to the complementary properties of these two biopolymers, as previously demonstrated in edible film formation [59]. Collagen gels are typically soft and elastic, with limited thermal stability [45], whereas agar can improve gel strength and thermal resistance [53]. Therefore, compared with single-component fat replacers, collagen–agar systems may provide more balanced functionality by combining protein-derived emulsifying properties and desirable textural characteristics with improved structural stability during thermal processing. These features make collagen–agar systems suitable candidates for fat replacement in thermally processed meat products. A previous study demonstrated the potential of a collagen–agar emulsion gel (CAEG) in meat product applications [60]. The CAEG was used as a partial replacer for pork jowl in a meatloaf model system, with up to 50% replacement achieving a favorable balance between technological functionality and nutritional quality. However, that study was conducted on products derived from a raw meat matrix, in which myofibrillar proteins retain their functional properties. In contrast, liver pâté-type products are produced from fat and meat raw materials that undergo thermal pretreatment (typically scalding), resulting in protein denaturation and a fundamentally different structural matrix [4,5]. These differences may significantly influence interactions between emulsion gel systems and the meat matrix, particularly regarding fat and water binding, as well as texture development. Therefore, the applicability of CAEG in pâté-type systems requires separate investigation.

Therefore, the aim of the present study was to evaluate the effect of replacing animal fat with an emulsion gel composed of rapeseed oil, pork collagen, and agar on selected quality parameters in model liver pâté systems.

## 2. Materials and Methods

### 2.1. Materials

Pork collagen powder (91–94% protein; <3% fat) was provided by ALL SPICE Niegodzisz sp. j. (Dziekanów Nowy, Poland). Agar powder (agarose 70%; agaropectin 30%) was purchased from Biomus sp. z o.o. (Lublin, Poland). Pork shoulder and pork jowl were obtained from Sokołów S.A. (Sokołów Podlaski, Poland), and pork liver from Zakłady Mięsne Skiba S.A. (Chojnice, Poland). A broth was obtained from the scalding of pork shoulder in water. Refined rapeseed oil (saturated fatty acids 6.4%; monounsaturated fatty acids 59.1%; polyunsaturated fatty acids 25.5%) was supplied by Komagra sp. z o.o. (Kosów Lacki, Poland). Fine table salt was purchased from Cenos Sp. z o.o. (Września, Poland), and ground black pepper from Prymat sp. z o.o. (Jastrzębie-Zdrój, Poland).

The following analytical-grade reagents were used: trichloroacetic acid (≥99% purity) and 2-thiobarbituric acid (≥98% purity), both supplied by Sigma-Aldrich (Bellefonte, PA, USA).

### 2.2. Preparation of Emulsion Gel

The emulsion gel was formulated based on the literature data [57,61,62] and preliminary experiments demonstrating that agar improved the stability of collagen-based emulsion gels. As shown in Table S1 (Supplementary Materials), the CAEG exhibited significantly (*p* < 0.05) higher oil-binding capacity and hardness compared to the CEG, both in the primary state (after first gelation) and in the secondary state (after heat treatment followed by re-gelation).

The CAEG was prepared according to the composition presented in Table 1. Emulsion gel production comprised three stages: preparation of the aqueous and oil phases, formation of the O/W emulsion (50/50, *w*/*w*), and subsequent gelation of the emulsion. First, a 1% (*w*/*w*) agar solution and a 10% (*w*/*w*) pork collagen solution were prepared separately. The agar solution was heated to approximately 95 °C, while the collagen suspension was prepared in distilled water at room temperature. Both solutions were then equilibrated in a water bath MILL 147 (AJL Electronics, Krakow, Poland) at 60 °C for 10 min and mixed to obtain the collagen–agar aqueous phase. The mixture was maintained at 60 °C for 60 min with intermittent stirring. After 40 min, rapeseed oil was preheated at 60 °C for 20 min. Once both phases reached 60 °C, the collagen–agar aqueous phase and the oil phase were immediately emulsified. Emulsification was performed by homogenizing the aqueous phase (collagen–agar) while gradually adding the oil phase using a mechanical homogenizer (SilentCrusher M, Heidolph Scientific Products GmbH, Schwabach, Germany), at 15,000 rpm for 2 min. The resulting emulsions were deaerated by a vacuum sealer, Multivac C200 (Multivac, Wolfertschwenden, Germany), by applying two pressure cycles down to about 70 Pa. Finally, emulsions were stored at 4–6 °C for about 3 h to allow gelation and structural stabilization prior to further application. Approximately 200 g of emulsion gel was produced per batch.

### 2.3. Production of Model Liver Pâté-Type System

Model meat systems (batters and blocks) were prepared according to the recipe composition presented in Table 2.

As is typical for edible offal processed meat products, pork shoulder and jowl were scalded separately in water at approximately 85 °C until a soft and semi-soft consistency was achieved, respectively. The remaining broth from scalding the pork shoulder was collected as one of the ingredients for the production of model pâté systems [5,6]. The raw materials were then ground using a MESKO AL2-1-type grinder (MESKO-AGD Sp. z o.o., Skarżysko-Kamienna, Poland) and a grinding plate with 4.5 mm-diameter holes. Their composition was standardized using a FoodScan™ 2 Lab analyzer (FOSS Analytical A/S, Hillerød, Denmark). Table S2 (Supplementary Materials) presents the proximate composition of the scalded pork shoulder and pork jowl. The scalded pork shoulder contained approximately 60% water, 10% fat, 4% SFA, and 17% protein, whereas the scalded pork jowl contained approximately 31% water, 58% fat, 30% SFA, and 11% protein. Raw pork liver was ground twice using a Zelmer ZMM4048B grinder (Zelmer S.A., Rzeszów, Poland) and a grinding plate with 2 mm-diameter holes and mixed with part of the salt. All components were weighed according to the recipe composition (Table 2), and two-stage chopping was performed in a Stephan UM 5 cutter (Stephan Machinery GmbH, Hameln, Germany) at 3000 rpm. In the first stage, scalded pork shoulder and jowl/CAEG, along with a broth from meat scalding, were chopped for 4 min at approximately 60 °C. In the second stage, liver, remaining salt, and black pepper were added, and the batter was chopped for an additional 4 min at approximately 40 °C.

Approximately 500 g of model pâté batter was prepared per batch, with about 150 g collected for analysis. The remaining portion (~300 g) was transferred into aluminum molds (189.5 × 85.5 × 50.5 mm; Alfatec Pro Sp. z o.o., Wysogotowo, Poland), and heat-treated in a convection–steam oven (Rational SCCWE61, Rational AG, Landsberg am Lech, Germany) at 180 °C under 0% humidity (airflow level: 3/5) until the geometric center reached 75 °C. Baked model pâtés were initially cooled to 30 °C using air-blast cooling in the oven. Subsequently, the model pâtés were transferred to a refrigerated cabinet (4–6 °C), protected from light exposure, and left uncovered for approximately 1 h to allow gradual cooling and prevent surface condensation. The samples were then wrapped in aluminum foil and stored under refrigerated conditions (4–6 °C) for 18–20 h to stabilize their final structure before measurements. All meat systems were prepared and analyzed in three independent batches.

### 2.4. Analyses of Model Liver Pâté Batters

#### 2.4.1. pH Level

The pH values of the model pâté batters were determined according to the reference method [63] using a Testo 206-pH2 pH meter (Testo SE & Co. KGaA, Germany) equipped with automatic temperature compensation. The device was calibrated prior to analysis with standard buffer solutions at pH 4.0 and 7.0. Approximately 10 g of sample was thoroughly mixed with 30 mL of distilled water in a 50 mL beaker. The suspension was left to stand for 10 min at 22 ± 1 °C, after which pH was measured by direct electrode immersion. Measurements were performed in triplicate.

#### 2.4.2. Color Parameters

The color of model pâté batters was evaluated using the reflectance method in the CIE Lab* color space [64]. Measurements were performed with a CR-400 colorimeter (Minolta Co., Osaka, Japan) equipped with a 2 mm aperture, D65 illuminant, and 2° standard observer. Prior to analysis, the instrument was calibrated using a white reference standard (Y = 95.1, x = 0.3159, y = 0.3326).

Samples were placed in transparent plastic Petri dishes (6 cm diameter, 1 cm height) of defined dimensions. Color coordinates (+L*: lightness; +a*/−a*: redness/greenness; +b*/−b*: yellowness/blueness) were recorded by applying the measuring head directly to the sample surface at five different positions.

In addition, the total color difference (ΔE) between sample types was calculated according to the following equation [65]:

(1) ΔE = √[(ΔL*)^2^ + (Δa*)^2^ + (Δb*)^2^],

where ΔL*, Δa*, and Δb* represent the differences in mean L*, a*, and b* values between the compared samples.

#### 2.4.3. Apparent Viscosity

The apparent viscosity of the model pâté batters was determined using a Rheotest-2 rotational viscometer (RHEOTEST Medingen GmbH, Ottendorf-Okrilla, Germany). A high-viscosity H/H measuring system, consisting of a stationary outer cylinder and a rotating inner cylinder, was applied. About 17 g of sample was introduced into the measuring chamber, and the α value was recorded after 30 s of operation [66]. Apparent viscosity (η) was subsequently calculated using the following equation:

(2) η = 0.1 (Z × α)/Dr

where Dr is the shear rate (16.2 s^−1^), Z is the instrument constant for the applied cylinder (294.3 dyn·cm^−2^·µs), and α is the value obtained from the viscometer (µs).

### 2.5. Analyses of Baked Model Liver Pâtés

#### 2.5.1. Cooking Yield

The cooking yields of the model pâtés were determined based on the differences in sample weight before and after heat treatment [67], according to the following equation:

(3) Cy = (m_2_/m_1_) × 100

where Cy is the cooking yield (%), m_1_ is the initial weight of the model pâté batter (g), and m_2_ is the weight of the model pâté after heat treatment and refrigerated storage (g).

#### 2.5.2. pH Level

The pH values of the baked model pâtés were determined in accordance with the standard [63] using a Testo 206-pH2 pH meter (see Section 2.4.1). Measurements were performed in triplicate for each sample.

#### 2.5.3. Water Activity

Water activity (a_w_) was determined according to the principles described in the ISO standard [68] using an AquaLab Series 3 m (METER Group Inc., Pullman, WA, USA). Ground samples were placed in sealed plastic containers and equilibrated for 120 min at 22.0 ± 0.5 °C prior to analysis. Measurements were performed in triplicate.

#### 2.5.4. Proximate Composition and Energy Value

The proximate composition and energy value of baked model pâtés were determined by near-infrared (NIR) transmission spectrometry (850–1100 nm) using a FoodScan™ 2 Lab analyzer integrated with ISIScan™ Nova software for FoodScan™ 2 Lab analyzer (FOSS Analytical A/S, Hillerød, Denmark). The instrument employs a global Artificial Neural Network (ANN) calibration model for proximate composition analysis [69]. Model pâté blocks were ground twice using a grinder equipped with a grinding plate with 3 mm-diameter holes. Approximately 200 g of the ground sample was carefully placed in an optical glass cup (140 mm diameter) and then inserted into the instrument chamber. The contents of water, fat, saturated fatty acids, protein, and collagen (mass%) were determined in accordance with the Polish standard PN-A-82109:2010 [70]. The energy value (kcal/100 g) was calculated based on the amounts of individual nutrients using the conversion factors specified in Annex XIV to Regulation (EU) No 1169/2011 of the European Parliament and of the Council [71]. Measurements were performed in triplicate for each sample.

#### 2.5.5. TBARS Index

The level of lipid oxidation in baked model pâtés was determined using the TBARS index, which reflects the concentration of thiobarbituric acid-reactive substances, mainly malondialdehyde (MDA) [72]. A 2.00 ± 0.01 g sample of ground pâté was weighed into a centrifuge tube. Then, 5 mL of 10% trichloroacetic acid (TCA) was added, and the sample was homogenized for 2 min. Subsequently, 5 mL of 0.02 M thiobarbituric acid (TBA) solution was added, and homogenization was repeated for 2 min. The samples were centrifuged at 4000 rpm for 10 min at 15 °C using a Sigma 4K15 centrifuge (Sartorius Polska Sp. z o.o., Kostrzyn, Poland). The supernatant was filtered through qualitative filter paper (Ahlstrom Oyj, Helsinki, Finland, 65 g/m^2^) into test tubes, covered with foil, and incubated in a boiling water bath (MILL 147, AJL Electronics, Zawiercie, Poland) for 35 min. After cooling under running cold water, the solutions were transferred to glass cuvettes (3.5 mL capacity, 10 mm optical path length), and absorbance was measured at 532 nm using a Camspec M501 single-beam spectrophotometer (Spectronic Camspec Ltd., Garforth, UK). The instrument was blanked using a reagent mixture containing 5 mL of 10% TCA and 5 mL of 0.02 M TBA. The TBARS value was calculated according to the following:

(4) TBARS = A_532_ × 2.34

where TBARS is an indicator of secondary lipid oxidation (mg MDA/kg of sample), A_532_ is the absorbance at 532 nm, and 2.34 is the conversion factor that accounts for sample dilution and analytical conditions.

#### 2.5.6. Shear Force

The shear force of baked model pâtés was determined instrumentally using a Zwicki 1120 universal testing machine and TestXpert II software for Zwicki 1120 (Zwick GmbH & Co. KG, Ulm, Germany). The measuring set consisted of a flat blade (the moving element) and a stationary platform with a slot adapted to the blade’s dimensions. Samples were prepared as 5 cm-long, 1 cm-wide slices. The maximum force (N) required to shear through the sample was recorded [73]. Measurements were performed at a crosshead speed of 2 mm s^−1^, with a preload of 0.2 N and a maximum load cell capacity of 50 kg [74]. All tests were conducted at room temperature (22 ± 2 °C). Each slice was cut at three different positions. Nine replicates were performed for each sample type.

#### 2.5.7. Microscopic Observation

The microscopic observation of sliced model pâtés was performed using a metallographic microscope (Delta Optical 100 TP) equipped with a DLT-Cam PRO 6.3 MP digital camera (Delta Optical, Mińsk Mazowiecki, Poland). For this purpose, a 1 cm-thick slice was cut from each pâté block using a sharp knife. The slice surface was gently blotted with a paper towel to remove excess moisture, then placed on the black microscope stage. Observations were performed in reflected light at 20× magnification. Digital micrographs, including a calibrated scale bar (10 μm), were captured using the compatible DLT-Cam Viewer software 3.7 (Delta Optical, Mińsk Mazowiecki, Poland). The images obtained were used for qualitative assessment of the structural characteristics of the pâté’s cross-sectional surface.

#### 2.5.8. Color Parameters

The color of the baked model pâtés was assessed using a CR-400 colorimeter (see Section 2.4.2). Measurements of L*, a*, and b* parameters were taken on the cross-section of pâté blocks sliced into 1 cm-thick samples. Each measurement was performed by placing the measuring head on the sample surface at five different locations [75]. ΔE values were then calculated, and the results were interpreted as for the model pâté batters (as detailed in Section 2.4.2).

### 2.6. Statistical Analyses

Statistical analyses were performed using Statistica 13.3 software (TIBCO Software Inc., Palo Alto, CA, USA). Differences among the analyzed model liver pâté systems were evaluated by one-way analysis of variance (ANOVA), with the replacement level of animal fat by emulsion gel as the grouping factor. The assumptions of normality and homogeneity of variance were verified using the Shapiro–Wilk and Brown–Forsythe tests, respectively. The significance level was set at α = 0.05. Differences were considered statistically significant at *p* < 0.05. Homogeneous groups were identified using Tukey’s HSD post hoc test. Moreover, Student’s *t*-test was used to compare the mean values of a given parameter between two groups (e.g., pH values of model batters and baked pâtés prepared with the same level of animal fat replacement) [76].

## 3. Results and Discussion

### 3.1. The Effect of Replacing Scalded Pork Jowl with Collagen–Agar Emulsion Gel on pH, Color Parameters, and Apparent Viscosity of Model Pâté Batters

The pH is a key quality parameter of semi-finished and finished processed meat products, influencing their technological properties as well as microbiological safety [77,78]. Table 3 presents the mean pH values of the model pâté batters, ranging from 6.2 to 6.3. Such values are typical for this type of semi-finished product and can be attributed to the raw materials used, namely, raw pork liver [79], as well as scalded fat raw materials. The incorporation of the emulsion gel into the formulation, regardless of the level of scalded jowl replacement, did not significantly affect the pH of the model batters. This may be explained by the fact that the CAEG exhibited a pH above 6.0, similar to that of the scalded pork jowl (see Tables S1 and S2, respectively, in the Supplementary Materials).

The available literature reports varying effects on the pH value of replacing animal fat in meat products, depending on the type of replacer used. Choi et al. [80] observed an increase in pH when pork fat was replaced with an emulsion of vegetable oils and rice bran fiber. In contrast, Serdaroğlu et al. [81] reported a decrease in pH after partial or complete replacement of beef fat with a gelled emulsion based on olive oil, inulin, and gelatin.

The color of meat batters and meat products is significantly influenced by factors such as pH, degree of comminution, fat content, the presence of functional ingredients, and processing conditions, including heat treatment and batter aeration [82]. The color parameters L*, a*, and b* of the model pâté batters are presented in Table 3. Statistical analysis revealed no significant differences (*p* > 0.05) among the pâté batter formulations for any of the color parameters analyzed. Furthermore, based on the L*, a*, and b* values, the total color difference between model pâté batters containing CAEG and the control batter (ΔE_R0_) was calculated. For the sample with 25% jowl replacement, ΔE_R0_ was <1.0, indicating no perceptible color differences compared with the control. For the remaining samples, ΔE_R0_ ranged from 1.0 to 2.0 (Table 3), suggesting that color differences would be noticeable only to an experienced observer [83]. The appearance of the pâté batters is shown in Figure S1 (Supplementary Materials). According to Domínguez et al. [84], color changes in meat systems subjected to animal fat replacement depend on the type, composition, and level of incorporated replacers. Moreover, color differences may be less pronounced in homogenized batters than in systems with a lower degree of comminution (e.g., semi-coarsely or coarsely comminuted).

Apparent viscosity is defined as a measure of a material’s resistance to flow at a given shear rate. It is commonly used to characterize the rheological behavior of non-Newtonian systems such as suspensions, emulsions, gels, and pastes. In food systems, apparent viscosity depends on chemical composition (e.g., water, fat, and protein content) as well as microstructure, which is modified by mechanical treatments such as mixing, comminution, and shear processing [85].

**Figure 1:**
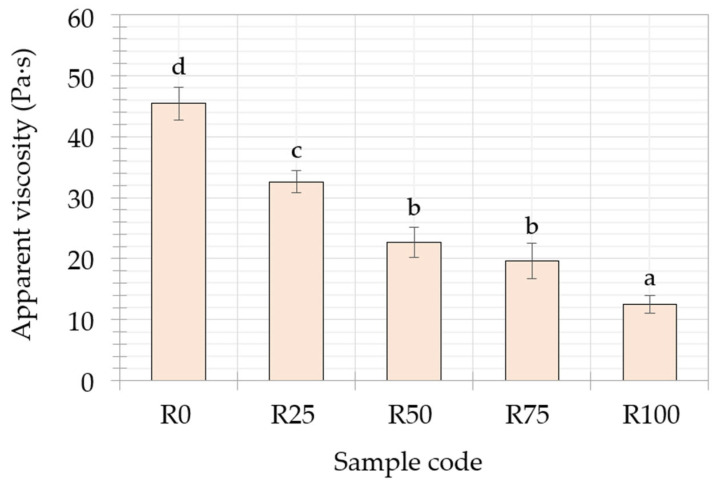
Apparent viscosity of model pâté batters. Explanation of the sample codes: R0/25/50/75/100—model pâté batters with 0/25/50/75/100% replacement of scalded pork jowl with collagen–agar emulsion gel (CAEG). Different lowercase letters (a–d) indicate significant differences between model pâté batters (*p* < 0.05). Exact *p*-values for the statistical comparisons are provided in Table S3 of the Supplementary Materials.

Figure 1 shows the apparent viscosity of the model pâté batters, which ranged from 12.5 Pa·s to 45.4 Pa·s. Replacing pork jowl fat with emulsion gel significantly (*p* < 0.05) reduced batter viscosity. Formulations with 50% and 75% pork jowl replacement showed approximately two-fold lower values compared with the control, while the lowest viscosity was observed in the batter with complete animal fat replacement. These findings are consistent with Woo et al. [39], who reported that replacement of pork fat with a corn oil-based emulsion gel containing polysaccharides (agar, gum arabic, and konjac glucomannan) significantly reduced the apparent viscosity of homogenized meat batters. The authors attributed this effect to reduced adsorption of myofibrillar proteins, particularly myosin, at the surface of emulsion gel particles compared with animal fat, related to differences in interfacial polarity.

According to Kim et al. [86], a reduction in the apparent viscosity of meat batters may compromise system stability during thermal processing, leading to increased weight loss and a weakened final product structure. Therefore, low-viscosity batters may require additional technological adjustments, such as modified mixing conditions or the incorporation of stabilizing agents.

### 3.2. The Effect of Replacing Scalded Pork Jowl with Collagen–Agar Emulsion Gel on Cooking Yield, pH, and Water Activity of Baked Model Pâtés

Cooking yield is an important indicator of technological efficiency in meat processing, reflecting weight losses (mainly water, fat, and soluble matter) during heating, as well as structural changes in meat, fat, and connective tissues [87,88]. As shown in Table 4, the model pâtés exhibited cooking yields ranging from 90% to 94%. The incorporation of CAEG as a scalded jowl replacer had no significant effect (*p* > 0.05) on this parameter. The literature indicates that stable homogenized meat systems typically have cooking yields exceeding 90% [86]. Serdaroğlu and Karaman [89] reported a significant reduction in the cooking yield of poultry pâtés, compared to the control sample, when beef was partially or completely replaced with a sunflower oil-based oleogel structured with carnauba wax. In contrast, Nacak et al. [90] found that 50% replacement of beef fat with an emulsion gel based on vegetable oils (peanut and linseed), inulin, egg white, gelatin, and PGPR significantly increased the cooking yield of homogenized beef sausages.

Based on the results presented in Table 4, the model pâtés with emulsion gel did not differ significantly (*p* > 0.05) from the control sample in terms of mean pH values. Moreover, the pH values of all model pâtés were comparable (Table S4) to those of the corresponding batters (Table 3). Similar findings were reported by Zając et al. [91], who observed no significant effect on pH in pâtés with 50% substitution of animal fat by an O/W emulsion based on olive oil and β-glucan. In contrast, Botella-Martínez et al. [92] demonstrated that the level of emulsion gel replacement significantly influenced pâté pH; no change was observed at 10% substitution, whereas increasing it to 20% resulted in a decrease in pH compared with the control sample. Likewise, Martins et al. [93] reported a reduction in pH following 60% replacement of pork fat with a linseed oil-based oleogel containing beeswax.

Water activity is a physicochemical parameter of food that describes the availability of water in a product and its involvement in chemical and biological reactions. An appropriate level of water activity is crucial for the microbiological safety and shelf life of the final product, as its reduction limits the growth and activity of microorganisms, including pathogens [94].

The baked model pâtés showed no significant differences (*p* > 0.05) in water activity, which was approximately 0.97, irrespective of recipe composition (Table 4). Comparable results were reported by Florowski et al. [5], who investigated the effect of walnut paste addition on selected quality parameters of liver sausage. According to Pałacha and Makarewicz [95], meat products have water activity values ranging from 0.918 to 0.990. The authors emphasized that edible offal processed meat products (e.g., sausages and pâtés) belong to meat products with particularly high water activity, typically above 0.98. Baranowska et al. [96] attributed the high water activity in baked pâtés to their specific composition and structure, in which a substantial proportion of water remains in a free or weakly bound state.

Accordingly, owing to their high water activity (0.968–0.970) and near-neutral pH (6.24–6.31), the model pâtés should be regarded as highly perishable products. However, a specific shelf life cannot be proposed for the present formulations because microbiological quality, sensory acceptability, and physicochemical stability were not evaluated during storage. The shelf life of such products may depend on several factors, including the microbiological quality of the raw materials, the effectiveness of heat treatment, the extent of post-process contamination, the packaging system, and the storage temperature. For further product development, rapid cooling after heat treatment, hygienic post-process handling, immediate packaging in materials with suitable gas- and moisture-barrier properties, and uninterrupted refrigerated storage at 0–4 °C should be considered. Vacuum packaging or an appropriately selected CO_2_-containing modified-atmosphere system may be suitable options; however, their effectiveness should be verified experimentally, as the packaging atmosphere can influence microbial growth, lipid oxidation, package integrity, and the product’s structural and sensory properties. Therefore, the optimal packaging system and use-by period should be established separately for each formulation through dedicated refrigerated shelf-life studies, including microbiological, physicochemical, oxidative, and sensory analyses and, where appropriate, microbiological challenge testing.

### 3.3. The Effect of Replacing Scalded Pork Jowl with Collagen–Agar Emulsion Gel on Proximate Composition, Energy Value, and TBARS Index of Baked Model Pâtés

Edible offal processed meat products exhibit diverse chemical compositions depending on the raw material characteristics, recipe composition, and technological processes used. The content and quality of individual components often substantially influence the nutritional value of the final product [97,98,99].

In the present study, the baked model pâtés were characterized in terms of proximate composition, including water, fat, saturated fatty acids (SFA), protein, and collagen content, as well as energy value. The results are presented in Table 5. The incorporation of CAEG as a jowl replacer significantly affected the composition of the model pâtés. Increasing the replacement level increased water content from approximately 51% to 56%. The R25 and R50 pâtés exhibited approximately two- and nearly three-fold higher collagen contents, respectively, compared with the control (~1.1%). However, further increases in CAEG incorporation did not significantly affect collagen content (*p* > 0.05).

The R25 formulation did not differ significantly from R0 in protein, fat, or SFA content. In contrast, ≥50% replacement of scalded jowl resulted in significant (*p* < 0.05) reductions in these components. Complete replacement of animal fat with CAEG reduced both fat and SFA contents in baked model pâtés by approximately 4 percentage points compared with R0. Consequently, energy value decreased, with R75 showing a 10% lower energy value than the control sample (Table 5). These changes were mainly attributed to differences in the composition of CAEG (Table 1) and scalded jowl (Table S2).

Overall, the effects of animal fat replacement in emulsified meat systems depend on both the level of replacement and the composition of the fat replacer. For example, Cîrstea (Lazăr) et al. [100] demonstrated that 50% replacement of pork backfat with emulsion gels based on a mixture of oils (chia seed, algae, and olive oil), stabilized with soy protein isolate and gelatin or chitosan, significantly increased water content and reduced fat and energy content in pork pâtés compared with the control product. However, no significant changes in protein content were observed. Similarly, de Souza Paglarini et al. [101] reported that partial (50%) replacement of pork backfat with an emulsion gel composed of soybean oil, soy protein, chia seed flour, sodium caseinate, carrageenan, sodium triphosphate, and inulin did not significantly affect water, protein, or fat content in Bologna-type sausages. An increase in water content and a decrease in fat content were observed in the sausages in which an emulsion gel completely replaced the lard, compared to sausages made only with this animal fat. In contrast, Domínguez et al. [102] found no significant effects of lard replacement with olive oil on water, protein, or fat content in pork pâtés.

The secondary stage of lipid oxidation involves the decomposition of previously formed hydroperoxides, leading to the formation of low-molecular-weight compounds such as aldehydes and ketones. These compounds are responsible for the development of undesirable odor and flavor associated with rancid fats in food products, including meat products. The level of secondary lipid oxidation products, commonly determined using the TBARS assay, reflects the extent of quality deterioration and reduced shelf life resulting from various technological factors [13,103].

**Figure 2:**
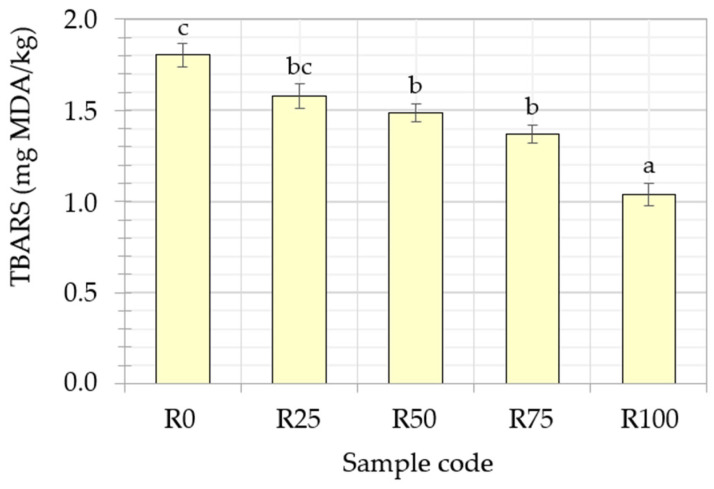
TBARS index of baked model pâté. Explanation of the sample codes: R0/25/50/75/100—baked model pâtés with 0/25/50/75/100% replacement of scalded pork jowl with collagen–agar emulsion gel (CAEG). Different lowercase letters (a–c) indicate significant differences between model pâté batters (*p* < 0.05). Exact *p*-values for the statistical comparisons are provided in Table S3 of the Supplementary Materials.

Mean TBARS values for the baked model pâtés ranged from 1.0 mg MDA/kg to 1.8 mg MDA/kg. Replacing pork jowl with CAEG at 25% had no significant effect (*p* > 0.05) on TBARS levels. In contrast, increasing the replacement level to 50% and 75% resulted in a significant reduction in TBARS values (by approximately 20%) compared with the control model pâté. Moreover, the R100 sample exhibited the lowest TBARS value among all tested model pâté variants (Figure 2). Similar findings were reported by Serdaroğlu et al. [104], who applied an emulsion gel based on flaxseed oil, structured with inulin and gelatin, as a partial replacement for beef fat in model homogenized meat systems. In contrast, Botella-Martínez et al. [105] reported different results, showing that partial replacement (25–50%) of pork backfat with an emulsion gel formulated with amaranth flour and chia seed oil led to a statistically significant increase in TBARS values in beef burgers compared with the control.

The observed decrease in TBARS values in the model pâtés with at least 50% of the animal fat was likely due primarily to their lower total fat content (Table 5). This is consistent with studies by Lorenzo et al. [106] and Lazárková et al. [11], who reported that TBARS values decreased significantly with decreasing fat content in pâtés. However, Skałecki et al. [9] obtained pork pâtés with markedly higher TBARS values (approximately 2.8 mg MDA/kg product) than those observed in the present study (Figure 2), despite a lower fat content (approximately 20%). In contrast, Terrasa et al. [107] produced poultry–pork pâtés with a comparable fat content (approximately 27%) but substantially lower TBARS values (approximately 0.3 mg MDA/kg product). These differences, however, may be attributed to the use of functional additives like phosphates, sodium nitrite, and ascorbic acid, which help inhibit lipid oxidation in meat products.

It should be emphasized that the TBARS threshold associated with the onset of consumer rejection is not universal and may vary depending on the type of raw material, fat content, individual consumer sensitivity, and the analytical method used [108,109].

Furthermore, increasing CAEG content in the model pâtés led to a reduction in SFA content (Table 5), suggesting a potential increase in the lipid fraction’s susceptibility to oxidation. However, this was not reflected in the TBARS values measured 1 day after production of the model liver pâtés, indicating that lipid oxidation was in its early stage. The effect of fatty acid composition on lipid oxidation may become more noticeable during storage [13,110]. In addition, incorporation of rapeseed oil in the form of an emulsion gel may limit oxygen diffusion and reduce the availability of pro-oxidant compounds, thereby delaying the onset of oxidative reactions [33,111].

### 3.4. The Effect of Replacing Scalded Pork Jowl with Collagen–Agar Emulsion Gel on the Shear Force, Structure, and the Color Parameters of Baked Model Pâtés

Texture is a fundamental quality attribute of meat products, as it significantly influences consumer acceptance [112]. The textural characteristics of these products are influenced by pre-slaughter factors (e.g., species, age, and animal nutrition), post-slaughter factors (e.g., biochemical changes and cooling conditions), and technological factors (e.g., formulation and heat treatment parameters) [113,114,115]. One objective method for evaluating meat texture is the shear force test, which allows instrumental determination of product hardness and tenderness. Higher shear force values indicate increased sample hardness (decreased tenderness), which is often associated with greater perceived toughness [115,116].

**Figure 3:**
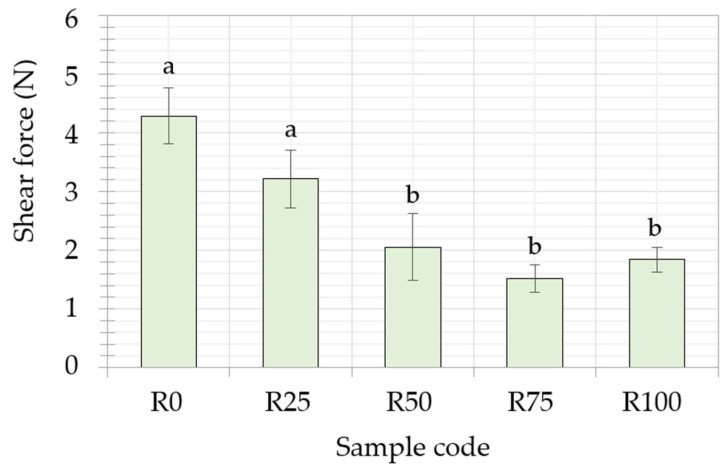
Shear force of sliced baked model pâté. Explanation of the sample codes: R0/25/50/75/100—baked model pâtés with 0/25/50/75/100% replacement of scalded pork jowl with collagen–agar emulsion gel (CAEG). Different lowercase letters (a, b) indicate significant differences between model pâté batters (*p* < 0.05). Exact *p*-values for the statistical comparisons are provided in Table S3 of the Supplementary Materials.

Figure 3 presents the mean shear force values for the model pâtés, ranging from 1.5 N to 4.3 N. Replacing 25% of the scalded pork jowl with CAEG did not significantly affect this parameter (*p* > 0.05) compared with R0. However, ≥50% replacement of animal fat resulted in a significant (approximately two-fold) reduction in shear force. These results are consistent with the apparent viscosity results of the model pâté batters (Figure 1), suggesting a relationship between meat batter consistency and final product hardness after heat treatment. These structural changes suggest that baked liver pâtés with ≥50% replacement of scalded jowl with CAEG may be perceived as less tough compared to those without the replacer.

Tiensa et al. [97] and Lazárková et al. [11] also reported that reducing the animal fat content decreases the instrumental hardness of pâtés. Similarly, Botella-Martínez et al. [92] found that replacing animal fat with an emulsion gel based on hemp oil, buckwheat flour, and gelatin significantly reduced the hardness of pork pâtés, despite an increase in total fat content.

**Figure 4:**
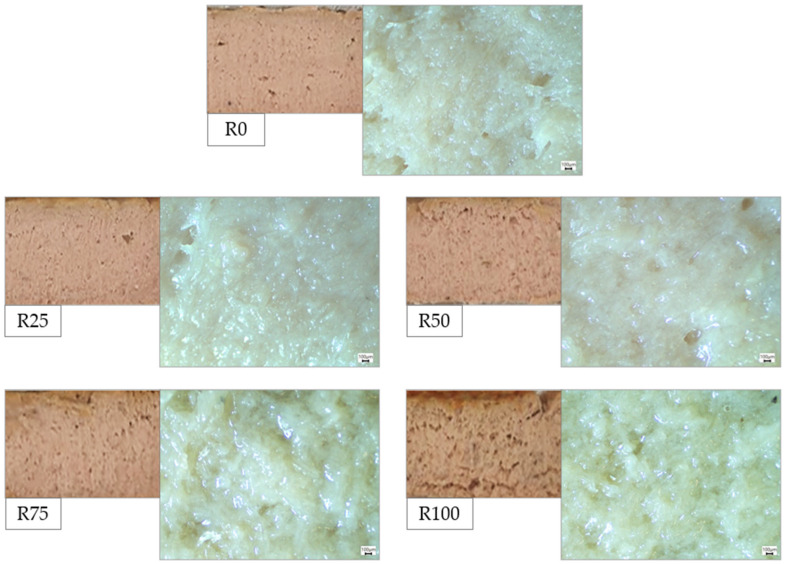
Cross-sectional images of the baked model pâtés and corresponding micrographs at 20× magnification. Explanation of the sample codes: R0/25/50/75/100—baked model pâtés with 0/25/50/75/100% replacement of scalded pork jowl with collagen–agar emulsion gel (CAEG).

A visual assessment of the cross-sectional structure of the model baked pâtés revealed significant structural variations among the tested formulations (Figure 4). The control sample (R0) exhibited the most compact and uniform structure. With increasing replacement of scalded pork jowl with CAEG, a reduction in ingredient binding and structural compactness was noted. Microscopic analysis indicated a progressive weakening of the fibrous network as the proportion of pork jowl decreased. In particular, samples R75 and R100 showed a less coherent, more porous structural matrix, along with localized accumulations of the liquid phase.

The observed structural and textural changes in the baked model pâtés may be explained by differences in the composition of the meat systems. The addition of CAEG to the model pâtés increased water content and decreased protein content, while increasing collagen content (Table 5). This indicates a lower proportion of myofibrillar proteins, which are important for forming the gel structure in meat products such as liver pâtés [117].

In addition, the reduction in fat content was accompanied by a decrease in SFA content, i.e., hydrophobic compounds can promote the formation of a more rigid crystalline fat structure [118]. A stable network of myofibrillar proteins that surrounds finely and evenly distributed fat particles, with low porosity, improves the cohesion and structural integrity of pâtés [97]. On the other hand, larger fat particles can weaken the protein–fat matrix, leading to a softer texture [119]. However, Yessimbekov et al. [99] showed that such pâté texture does not necessarily reduce consumer acceptability, as higher spreadability of these products was associated with higher organoleptic scores.

Thermal processing induces a series of physical and chemical transformations that significantly influence the quality attributes and consumer acceptance of meat products. Among the most evident effects of these transformations are changes in the overall appearance and color of the final products [2]. The color parameters of the model baked pâtés are presented in Table 6. Increasing the level of scalded jowl replacement with CAEG resulted in a slight decrease in the L* (from approximately 66.0 to 63.8) and a* (from approximately 6.8 to 5.5) values; however, these differences were not statistically significant. In contrast, samples with 75% and 100% replacement of this animal fat exhibited significantly higher b* values (*p* < 0.05) compared to the control sample. Similar findings were reported by Delgado-Pando et al. [67], who observed comparable color changes in reduced-fat pork pâtés when pork backfat was partially (50%) replaced with an O/W emulsion based on fish, linseed, and olive oils. Domínguez et al. [102] also reported increased yellowness in pork pâtés after 50% and 75% substitution of pork backfat with fish oil. Similarly, Martins et al. [93] found that a 30% replacement of pork fat with a linseed oil-based oleogel containing 8% beeswax increased the b* value, without affecting L*.

Additionally, the mean L* and b* values of the model pâtés (Table 6) did not differ substantially from those of the corresponding batters (Table 3). This may be explained by the fact that the meat and fat raw materials were subjected to a preliminary heat treatment, which established their color before the final thermal processing (baking). Consequently, baking did not lead to pronounced changes in lightness or yellowness. In contrast, heat treatment resulted in a significant (approximately 30%) reduction in the a* value of the pâtés compared with the model batters. The model pâté batters were prepared using raw pork liver, which contains red heme pigments (hemoglobin, myoglobin, and cytochromes). During heating, heme proteins denature, and myoglobin undergoes oxidation, resulting in a brownish-gray color and a reduction in the a* value of the meat systems [120]. The absence of curing agents (e.g., nitrites) prevents the formation of nitrosylmyoglobin, which, upon heat treatment, is converted into nitrosylhemochrome, responsible for the characteristic pink-red color of cured meat products [121].

Table 6 also presents the mean values of the total color difference between pâtés containing emulsion gel and the control sample (ΔE_R0_). The results indicate that visual differences in cross-sectional color between pâtés with 25%, 50%, or 75% pork jowl replacement and the control sample (1.0 < ΔE < 2.0) would be perceptible only to experienced observers. In contrast, only complete replacement of pork jowl resulted in color differences (2.0 < ΔE < 3.5) that could also be detected by untrained observers [83].

Both instrumental color measurements and visual perception may have been influenced not only by pigment transformations but also by structural differences in the model pâtés (Figure 4). The optical properties of food products, including light reflection, scattering, and refraction, depend on their microstructure and matrix homogeneity. A smooth, homogeneous surface promotes uniform light reflection, whereas irregular structures with pores and discontinuities increase light scattering. These differences may affect both measured color parameters and visual color perception [122].

From a practical perspective, CAEG presents both potential advantages and limitations compared with other structured fat-replacer systems. Because it contains a substantial aqueous phase, its incorporation may reduce not only saturated fatty acid content but also total fat content and energy value. Its preparation involves heating, homogenization, vacuum deaeration, and refrigerated gelation; however, the present study was conducted on a laboratory scale, and the scalability and cost-effectiveness of the process were not evaluated. Therefore, comparisons with oleogels in terms of production cost and industrial feasibility cannot be made without a dedicated techno-economic assessment. Oleogel production methods also differ considerably depending on the structuring agent; for example, beeswax-based oleogels require heating and subsequent cooling, whereas hydrocolloid-based oleogels may involve indirect structuring procedures [93,123].

The sensory impact of CAEG also remains uncertain because no sensory analysis was conducted in the present study. Previous studies have shown that the effect of structured lipid systems on sensory quality depends on their composition and replacement level. Partial replacement frequently produced products with sensory properties close to those of the controls, whereas higher replacement levels were more likely to affect texture, color, flavor, or consumer preference [61,62,90,93,123,124]. Storage stability is similarly formulation dependent. The lower TBARS values observed one day after production in the present study cannot be interpreted as evidence of improved stability throughout storage. Previous studies reported either maintained stability, no compromise in lipid and protein oxidation, or accelerated oxidation after incorporating structured vegetable-oil systems [61,62,90,91,92]. Consequently, sensory evaluation and refrigerated storage studies are required to determine the oxidative, structural, microbiological, and sensory stability of CAEG-containing pâtés.

## 4. Conclusions

The present study demonstrated that a collagen–agar emulsion gel (CAEG) containing rapeseed oil can be used as a partial replacer of scalded pork jowl in model liver pâté-type systems. The incorporation of CAEG did not adversely affect pH, cooking yield, water activity, or most color parameters of either the pâté batters or the baked pâtés. Moreover, replacing pork jowl with CAEG reduced the fat and saturated fatty acid contents and the energy values of the final products, while higher replacement levels were also associated with lower TBARS values, indicating lower levels of secondary lipid oxidation products in the early post-production period.

However, progressively increasing the replacement level altered the products’ technological characteristics. Model batters containing CAEG exhibited lower apparent viscosity, and pâtés obtained with ≥50% replacement showed reduced shear force and a less compact microstructure. These changes also suggest a weakening of the structural integrity of the pâté matrix. Considering the balance between the reduction in fat content and energy value and the maintenance of desirable technological properties, partial replacement of scalded pork jowl with CAEG, particularly at levels up to 50%, appears to be the most suitable reformulation strategy for liver pâté-type products.

Nevertheless, several limitations of this study should be acknowledged. The experiments were conducted using model pâté systems and focused on selected physicochemical and technological parameters evaluated one day after production. Sensory evaluation, consumer acceptance studies, chromatographic fatty acid profiling, and storage experiments assessing oxidative and microbiological stability were beyond the scope of the present work. Consequently, further studies should investigate the sensory quality and shelf life of CAEG-based liver pâtés during refrigerated storage and determine the packaging system that provides the most favorable microbiological, oxidative, structural, and sensory stability.

## Figures and Tables

**Table 1:** Composition of collagen–agar emulsion gel.

Component	Phase Type	Phase Mass Fraction (%)	Concentration (%)	
			In the Phase	In the Emulsion Gel
Water	Aqueous	50	94.5	47.25
Collagen			5.0	2.50
Agar			0.5	0.25
Rapeseed oil	Oil	50	100.0	50.00

**Table 2:** Recipe composition of model liver pâté-type systems (in mass %).

Ingredient	R0	R25	R50	R75	R100
Basic Raw Materials					
Pork shoulder	45				
Pork jowl	45.0	33.75	22.5	11.25	–
CAEG	–	11.25	22.5	33.75	45.0
Pork liver	10				
Broth *	20				
**Seasonings** **					
Table salt	1.5				
Black pepper	0.1				

* in relation to the weight of basic raw materials; ** in relation to the weight of basic raw materials and broth. Explanation of the sample codes: R0/25/50/75/100—model pâté systems with 0/25/50/75/100% replacement of scalded pork jowl with collagen–agar emulsion gel (CAEG).

**Table 3:** pH value and color parameters of model pâté batters (mean value ± standard deviation).

Sample Code	pH	Color Parameters			
		L*	a*	b*	ΔE_R0_
R0	6.26 ± 0.06	66.8 ± 1.2	9.4 ± 1.6	10.9 ± 0.6	–
R25	6.24 ± 0.05	66.1 ± 0.8	9.3 ± 1.8	10.6 ± 0.5	0.7
R50	6.22 ± 0.04	65.4 ± 1.2	9.3 ± 1.6	10.8 ± 0.6	1.4
R75	6.24 ± 0.05	65.2 ± 1.1	9.1 ± 1.6	11.3 ± 0.1	1.7
R100	6.23 ± 0.05	65.1 ± 1.1	9.1 ± 1.9	11.2 ± 0.5	1.8

Explanation of the sample codes: R0/25/50/75/100—model pâté batters with 0/25/50/75/100% replacement of scalded pork jowl with collagen–agar emulsion gel (CAEG). L*—lightness, a*—redness, b*—yellowness, ΔE_R0_—total color difference compared to the R0 sample. No significant differences were observed between model pâté batters (*p* > 0.05). Exact *p*-values for the statistical comparisons are provided in Table S3 of the Supplementary Materials.

**Table 4:** Cooking yield, pH value, and water activity (a_w_) of baked model pâtés (mean value ± standard deviation).

Sample Code	Cooking Yield (%)	pH	a_w_
R0	93.7 ± 1.8	6.31 ± 0.04	0.968 ± 0.001
R25	92.9 ± 1.9	6.27 ± 0.04	0.969 ± 0.001
R50	92.9 ± 1.7	6.26 ± 0.03	0.970 ± 0.001
R75	92.2 ± 1.7	6.24 ± 0.04	0.970 ± 0.001
R100	90.3 ± 1.9	6.24 ± 0.05	0.969 ± 0.001

Explanation of the sample codes: R0/25/50/75/100—baked model pâtés with 0/25/50/75/100% replacement of scalded pork jowl with collagen–agar emulsion gel (CAEG). No significant differences were observed between model pâté batters (*p* > 0.05). Exact *p*-values for the statistical comparisons are provided in Table S3 of the Supplementary Materials.

**Table 5:** Proximate composition and energy value of baked model pâtés (mean value ± standard deviation).

Sample Code	Content (%)					Energy Value (kcal/100 g)
	Water	Fat	SFA	Protein	Collagen	
R0	50.9 ± 0.6 a	28.0 ± 1.1 d	11.7 ± 0.9 d	19.4 ± 0.4 c	1.1 ± 0.1 a	327.9 ± 9.6 c
R25	52.2 ± 0.4 b	26.8 ± 0.9 cd	10.3 ± 0.6 cd	19.0 ± 0.2 bc	2.3 ± 0.1 b	315.5 ± 8.4 bc
R50	53.7 ± 0.2 c	25.6 ± 0.7 bc	9.4 ± 0.3 bc	18.6 ± 0.4 ab	3.0 ± 0.1 c	304.1 ± 7.6 ab
R75	55.0 ± 0.2 d	24.2 ± 1.1 ab	8.5 ± 0.2 ab	18.1 ± 0.1 a	2.9 ± 0.3 c	290.2 ± 9.8 a
R100	56.3 ± 0.4 e	23.2 ± 0.6 a	7.4 ± 0.3 a	18.0 ± 0.2 a	2.6 ± 0.2 bc	282.1 ± 6.8 a

Explanation of the sample codes: R0/25/50/75/100—baked model pâtés with 0/25/50/75/100% replacement of scalded pork jowl with collagen–agar emulsion gel (CAEG). SFA indicates saturated fatty acids, and different lowercase letters (a–e) in the column indicate significant differences between baked model pâtés (*p* < 0.05). Exact *p*-values for the statistical comparisons are provided in Table S3 of the Supplementary Materials.

**Table 6:** Color parameters of baked model pâtés (mean value ± standard deviation).

Sample Code	Color Parameters			
	L*	a*	b*	ΔE_R0_
R0	65.9 ± 1.4	6.8 ± 0.8	10.8 ± 0.2 a	-
R25	64.8 ± 1.2	6.2 ± 1.3	10.8 ± 0.2 ab	1.3
R50	64.6 ± 1.1	6.2 ± 0.6	10.8 ± 0.3 ab	1.5
R75	65.0 ± 1.6	6.0 ± 0.7	11.6 ± 0.4 bc	1.6
R100	63.8 ± 2.0	5.5 ± 0.9	12.0 ± 0.4 c	2.8

Explanation of the sample codes: R0/25/50/75/100—baked model pâtés with 0/25/50/75/100% replacement of scalded pork jowl with collagen–agar emulsion gel (CAEG). L*—lightness, a*—redness, b*—yellowness, ΔE_R0_—total color difference compared to the R0 sample. Different lowercase letters (a–c) in the column indicate significant differences between baked model pâtés (*p* < 0.05). Exact *p*-values for the statistical comparisons are provided in Table S3 of the Supplementary Materials.

## Data Availability

The original contributions presented in this study are included in the article/Supplementary Materials. Further inquiries can be directed to the corresponding authors.

## Supplementary Materials

The following supporting information can be downloaded at https://www.mdpi.com/article/10.3390/foods15152609/s1. Figure S1: Visual appearance of model pâté batters; Table S1: Comparison of selected properties of collagen emulsion gel and collagen–agar emulsion gel; Table S2: Proximate chemical composition and the pH of scalded pork shoulder and pork jowl; Table S3: Summary of exact *p*-values obtained from one-way ANOVA comparisons of the analyzed parameters; Table S4: Results of Student’s *t*-test comparing pH values of model batters and baked pâtés.
