# Supplementary material for: Rapeseed Oil-Based Collagen–Agar Emulsion Gel as an Alternative Fat Raw Material in Model Liver Pâté-Type Meat Systems
Source: Foods. 2026 Jul 25;15(15):2609. doi: 10.3390/foods15152609 (PMC13464909; doi:10.3390/foods15152609)
Supplement: Supplementary file 1 [file foods-15-02609-s001.zip › foods-4431113-supplementary.pdf]

Supplementary Materials

TABLES

Table S1. Comparison of selected properties of collagen emulsion gel and collagen-agar emulsion gel

Parameter	CEG	CAEG	Statistical analysis	
	Primary state†		*p*	*t*
pH (–)	7.78 ± 0.04	7.75 ± 0.05	0.9865	−0.06
OHC (%)	81.1 ± 1.8	96.3 ± 2.2	0.0008	9.05
Hardness (N)	9.8 ± 0.7	12.3 ± 0.9	0.0010	4.75
L* (–)	90.3 ± 1.1	93.7 ± 1.0	0.0173	3.92
b* (–)	7.1 ± 0.3	5.5 ± 0.2	0.0015	−7.79
	Secondary state††			
pH (–)	7.79 ± 0.06	7.73 ± 0.05	0.9741	−0.08
OHC (%)	66.0 ± 2.0	82.5 ± 2.2	0.0006	9.67
Hardness (N)	4.3 ± 0.5	8.2 ± 0.5	0.0067	4.46
L* (–)	84.3 ± 1.0	91.6 ± 0.9	0.0007	9.47
b* (–)	8.1 ± 0.4	6.3 ± 0.3	0.0032	−6.33

Explanation of the sample codes: CEG – collagen emulsion gel; CAEG – collagen-agar emulsion gel; OHC – oil-holding capacity; L* – lightness, b* – yellowness; † obtained after storage at 4–6 °C for 20 h post-production; †† obtained after reheating at 75 °C for 30 min followed by re-gelation under the same conditions; $p < 0.05$ means statistically significant differences between mean values of a given parameter (t-Student test, α=0.05).

Table S2. Proximate chemical composition and the pH of scalded pork shoulder and pork jowl.

Raw material	Content (%)				pH (–)
	Water	Fat	SFA	Protein	
Shoulder	59.7 ± 0.5	10.5 ± 0.4	4.1 ± 0.4	17.1 ± 1.0	5.8 ± 0.5
Jowl	30.7 ± 1.3	58.4 ± 1.4	29.8 ± 0.4	10.9 ± 0.3	6.2 ± 0.7

SFA – saturated fatty acids.

Table S3. Summary of exact p-values obtained from one-way ANOVA comparisons of the analyzed parameters.

Parameter	p-value
Model pâté batters	
pH (–)	0.90087
L* (–)	0.31744
a* (–)	0.99951
b* (–)	0.72536
Apparent viscosity (Pa·s)	<0.00001
Baked model pâtés	
Cooking yield (%)	0.27721
pH (–)	0.26491
Water activity (–)	0.58053
Water content (%)	0.00000
Fat content (%)	0.00034
SFA content (%)	0.00003
Protein content (%)	0.00029
Collagen content (%)	0.00000
Energy value (kcal/100 g)	0.00039
TBARS (mg MDA/ kg)	0.00042
Shear force (N)	0.00006
L* (–)	0.56481
a* (–)	0.50886
b* (–)	0.00238

L* – lightness, a* - redness, b* – yellowness, SFA – saturated fatty acids; $p < 0.05$ means statistically significant differences between mean values of a given parameter (one-way ANOVA test, α=0.05).

Table S4. Results of Student's t-test comparing pH values of model batters and baked pâtés.

Sample code	Student's t-test results	
	t	p
R0	1.265	0.275
R25	0.695	0.525
R50	1.463	0.217
R75	0.212	0.842
R100	0.398	0.711

Explanation of the sample codes: R0/25/50/75/100 – model pâté systems with 0/25/50/75/100% replacement of scalded pork jowl with collagen-agar emulsion gel (CAEG); No significant differences were observed between model pâté batters and baked pâtés in terms of mean pH values ($p > 0.05$).

FIGURES

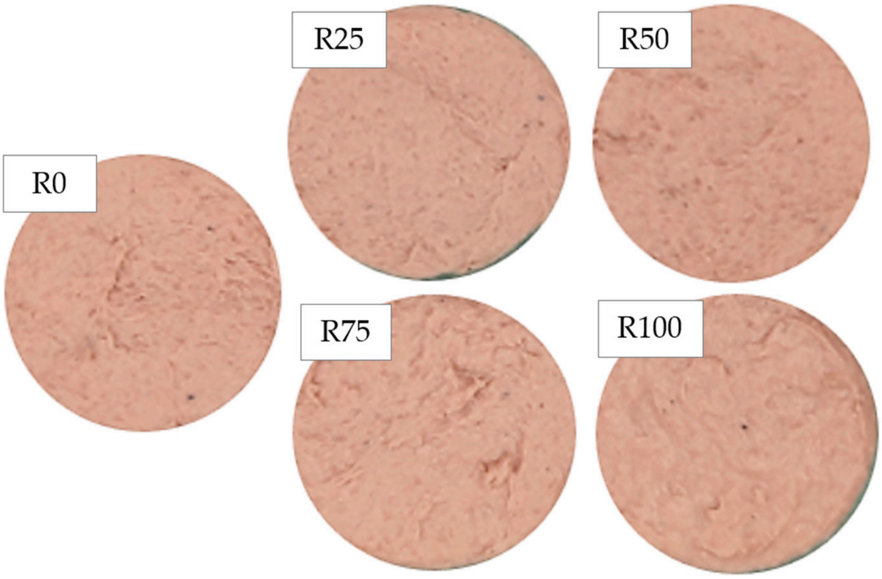

Figure S1. Visual appearance of model pâté batters.

Explanation of the sample codes: R0/25/50/75/100 – model pâté batters with 0/25/50/75/100% replacement of scalded pork jowl with collagen-agar emulsion gel (CAEG);
